# The differences of orthostatic hypotension in patients with Parkinson's disease and multiple system atrophy

**DOI:** 10.3389/fneur.2023.1070943

**Published:** 2023-01-26

**Authors:** Jingrong Zeng, Yingqi Xing, Shanshan Mei, Baolei Xu, Xiaofan Xue, Haixia Song, Erhe Xu

**Affiliations:** ^1^Department of Neurology, Xuanwu Hospital, Capital Medical University, Beijing, China; ^2^Department of Vascular Ultrasonography, Xuanwu Hospital, Capital Medical University, Beijing, China; ^3^Department of Neurology, Chaoyang Hospital, Capital Medical University, Beijing, China; ^4^Department of Neurology, The People's Hospital of Shijiazhuang, Shijiazhuang, Hebei, China

**Keywords:** Parkinson's disease, multiple system atrophy, orthostatic hypotension, active standing test, the ΔHR/ΔSBP

## Abstract

**Background:**

Multiple system atrophy (MSA) and Parkinson's disease (PD) have similar clinical presentations in their early stages. Orthostatic hypotension (OH) is a common autonomic dysfunction associated with MSA and PD. Heart rate (HR) and systolic blood pressure (SBP) changes are measured in response to the active standing test, which is widely used to screen for cardiovascular autonomic function.

**Objectives and methods:**

Overall, 255 patients (67 MSA, 188 PD) underwent continuous beat-to-beat non-invasive BP monitoring and active standing test. The total standing time was 10 min, and the BP differences between both groups were compared to determine whether the ΔHR/ΔSBP can differentiate both conditions.

**Results:**

Classical orthostatic hypotension (COH) (52%) and initial OH (19%) were most common in MSA and PD, respectively. MSA had a higher HR (75.0 ± 9.7 vs. 71.0 ± 10.7, *P* = 0.008) than PD in the supine position. SBP (135.70 ± 15.68 mmHg vs. 127.31 ± 15.14 mmHg, *P* = 0.106), diastolic BP (78.45 ± 12.36 mmHg vs. 67.15 ± 13.39 mmHg, *P* = 0.009) and HR (73.94 ± 8.39 bpm vs. 71.08 ± 13.52 bpm, *P* = 0.389) at baseline were higher in MSA-COH than in PD-COH. After adjusting for age and disease duration, the ΔHR/ΔSBP-10 min significantly discriminated MSA-COH from PD-COH (*P* = 0.031). An ΔHR/ΔSBP-10 min of 0.517 showed a sensitivity of 67% and specificity of 84% (AUC = 0.77, 95% CI: 0.63–0.91).

**Conclusion:**

The SBP, diastolic BP, and HR were higher in the supine position; however, ΔHR and ΔSBP were lower after standing in MSA patients than in PD patients. The ΔHR/ΔSBP-10 min discriminated between MSA-COH and PD-COH with quiet acceptable accuracy.

## 1. Introduction

Multiple system atrophy (MSA) and Parkinson's disease (PD) have similar clinical presentations in their early stages. Autonomic symptoms, such as orthostatic hypotension (OH) and urinary and erectile dysfunction, are the primary hallmarks of MSA. Research by International Parkinson and Movement Disorder Society has added neurogenic and symptomatic OH to the criteria for prodromal PD (1). OH is arguably the most common symptom of autonomic failure. It has individual negative effects on disability in α-synucleinopathies, reflecting the “malignant” phenotype of PD (2).

Based on the results of plasma norepinephrine levels, neuroimaging tests, and neuropharmacological tests (3), PD with OH present with loss of sympathetic innervation most noticeably in the heart. In contrast, patients with MSA have an intact cardiac sympathetic innervation (4). It is accepted that the lesion is normally postganglionic in PD, whereas it is preganglionic in MSA. A Δ heart rate (HR)/Δ systolic blood pressure (SBP)-3 min ratio of 0.492 bpm/mmHg was deemed to distinguish neurogenic OH from non-neurogenic OH; however, this index did not differ between the central and peripheral forms of autonomic dysfunction (5).

HR and SBP changes were measured in response to active standing, which is more widely used in clinical settings. Fanciulli et al. further studied neurogenic orthostatic hypotension (nOH) in PD and MSA patients and proved the ΔHR/ΔSBP represents a valuable nOH bedside screening, when tilt test facilities are not available (6). Continuous beat-to-beat non-invasive BP monitoring to analyze active stand testing can identify OH and its variants, including initial orthostatic hypotension (IOH), delayed recovery, classical orthostatic hypotension (COH), delayed orthostatic hypotension (delayed OH) (7). Although their pathophysiology has not been definitively established, the primary problem is that the distribution of all the variants remains unknown.

This study aimed to find the differences of BP in patients with MSA and PD and verify whether the ΔHR/ΔSBP can help in the differential diagnosis.

## 2. Materials and methods

### 2.1. Study population

We screened 255 consecutive patients with PD or MSA from the neurology ward of Capital Medical University Xuanwu Hospital in China between January 2021 and February 2022. The study was approved by the Ethics Committee of Capital Medical University Xuanwu Hospital. PD with clinical diagnosis or probable diagnosis and possible or probable MSA were diagnosed according to the Movement Disorder Society PD criteria (8) and second consensus diagnostic criteria for MSA (9). All the patients underwent the brain MRI to rule out other diagnoses. Moreover, (18)F-AV-133 PET was used to identification if necessary. Patients with atrial fibrillation, myocardial infarction, anemia, polypharmacy (defined as the current use of six or more different medications), varicose veins, infectious diseases, or malignancies were excluded from this study. All the patients didn't receive treatment for OH and patients with diabetes mellitus were well control (HbA1c < 7%). Those with severe intracranial and extracranial artery stenosis on transcranial Doppler sonography monitoring were excluded.

### 2.2. Clinical assessments

All demographic, clinical, and pharmacological data were recorded by specialists in movement disorders. The levodopa equivalent dose was calculated according to methods described in a previous study (10). The Parkinson's Disease Questionnaire 39 (PDQ-39) (11) and the Non-Motor Symptoms Scale (NMSS) (12) were used to evaluate the quality of life. The Mini-Mental State Examination (MMSE) and Montreal Cognitive Assessment-Beijing version (MoCA) were used to assess cognitive function.

### 2.3. Active standing test

The test was performed in a quiet room with an appropriate temperature between 21–23°C. The participants were asked to avoid alcohol, caffeine, and vigorous exercise and stop dopamine and vasoactive medications for 24 h before the examination (7). First, the patients were asked to lie supine for 5–10 min and then stand as quickly as possible without straining or arm movement. The disabled received assistance if needed. We used an EMS-9D PRO (Delica Medical, Shenzhen, China) to record non-invasive continuous blood pressure (NIBP) during the entire process. ΔSBP, Δ diastolic blood pressure (DBP), and ΔHR were calculated as the difference between baseline and subsequent time points, such as the instant (0s upon standing up completely), 30 s, 1 min, 3 min, 5 min, and 10 min. We divided the change in HR by the fall in SBP and expressed it as ΔHR/ΔSBP.

OH is classified as follows (7, 13):

1. IOH is defined as a maximum transient reduction in SBP of >40 mmHg and/or >20 mmHg in DBP within 15 s of standing in the absence of sustained OH. The drop of SBP recover to <20 mmHg and/or <10 mmHg in DBP 15–30 s after standing.
2. Delayed recovery is defined as delayed recovery of SBP to baseline values of more than 20 mmHg at 30 s of standing, but not meeting the criteria of COH.
3. COH is defined as a sustained decrease of ≥20 mmHg in SBP (or ≥30 mmHg in those with baseline hypertension) or ≥10 mmHg (≥15 mmHg in those with baseline hypertension) in DBP (or SBP <90 mmHg) occurring between 60 and 180 seconds of standing.
4. Delayed OH is defined as SBP/DBP fall ≥20/10 mmHg occurring first after 3 min of standing.
5. Orthostatic hypertension is defined as an increase of ≥20 mmHg in SBP or ≥10 mmHg in DBP (or >140/90 mmHg if patient is normotensive supine) occurring 60–180 s after standing.

### 2.4. Statistical analysis

Statistical analyses were performed using IBM SPSS v22, and 2-sided *P*-values < 0.05 was considered significant. Qualitative variables were reported by frequency (percentage) and compared with the Pearson χ*2* test (or Fisher exact test, where appropriate). Quantitative variables were expressed as mean (standard deviation) or median (interquartile range) tested for normal distribution with the Shapiro-Wilk test, and analyzed by Student's *t* test and the Mann–Whitney *U* test according to their distribution. To compare ΔBP, ΔHR, and ΔHR/ΔSBP between the diseases and different time points, ANOVA for repeated measurement was used and Bonferroni correction for multiple comparison between groups was applied using disease duration and age as covariates. Receiver operating characteristic curve analysis was performed to evaluate the accuracy, sensitivity, and specificity of ΔHR/ΔSBP in patients with PD and MSA.

## 3. Results

A total of 255 patients, 188 with PD (53.2% men and 46.8% women; mean age, 61.2 ± 11.7 years) and 67 with MSA (46.3% men and 53.7% women; mean age, 59.9 ± 6.9 years), were included in this study. There were no significant differences in age, sex, duration, and use of drugs such as antihypertensives and levodopa between the two disease groups. The MSA group had higher HR (75.0 ± 9.7 vs. 71.0 ± 10.7 bpm, *P* = 0.008) in the supine position and higher H-Y score [3(2,3) vs. 2(2,3), *P* = 0.000], NMSS scores (58.6 ± 49.2 vs. 33.3 ± 28.1, *P* = 0.001) and PDQ-39 (53.2 ± 36.9 vs. 34.4 ± 28.1, *P* = 0.002) than the PD group. However, Global cognitive performance, expressed by the MMSE and MoCA, was similar in both disease groups. The demographic and clinical data are summarized in Table 1.

**Table 1:** Demographic and clinical characteristics of PD and MSA.

	**MSA** **(*n =* 67)**	**PD** **(*n =* 188)**	***P***	**MSA-COH** **(*n =* 33)**	**PD-COH** **(*n =* 13)**	***P***
Age, y	59.9 ± 6.9	61.2 ± 11.7	0.284	59.8 ± 6.2	66.5 ± 15.4	0.152
Male sex, *n* (%)	31 (46.3%)	100 (53.2%)	0.330	19 (57.6%)	11 (84.6%)	0.101
H&Y stage	3 (2,3)	2 (1,2)	<0.001	3 (2,3)	2 (2,3)	0.240
Disease duration, y	2.4 ± 1.6	4.3 ± 3.8	0.398	2.3 ± 1.7	5.8 ± 3.7	0.006
Height, cm	165.6 ± 8.1	165.3 ± 8.2	0.813	166.7 ± 9.0	167.2 ± 9.4	0.879
Weight, kg	66.3 ± 11.8	66.3 ± 11.6	0.982	68.2 ± 10.6	62.5 ± 10.3	0.101
MSA-P, *n* (%)	40 (59.7%)			16 (48.5%)		
Education, *n* (%)			0.143			0.566
Junior high school and below	7 (10.4%)	34 (17.6%)		2 (6.1%)	2 (15.4%)	
Senior high school	28 (41.8%)	57 (30.3%)		16 (48.5%)	5 (38.5%)	
College and above	32 (47.8%)	97 (51.6%)		15 (45.5%)	6 (46.2%)	
Hypertension, *n* (%)	18 (26.9%)	66 (35.5%)	0.208	9 (27.3%)	2 (15.4%)	0.473
LEDD, mg	389.1 ± 429.1	418.6 ± 332.8	0.565	298.1 ± 51.9	367.8 ± 102.0	0.072
AHD, *n* (%)			0.447			0.185
CCB	8 (11.9%)	25 (13.3%)		3 (9.1%)	0 (0.0%)	
ACEI/ARB	5 (7.46%)	17 (9.04%)		1 (3.0%)	2 (15.4%)	
Beta blocker	1 (1.49%)	7 (3.72%)		0 (0%)	0 (0%)	
Diuretic	1 (1.49%)	9 (4.79%)		0 (0%)	0 (0%)	
Diabetes, *n* (%)	7 (10.4%)	34 (18.3%)	0.136	3 (9.1%)	4 (30.8%)	0.087
SBP-Base, mmHg	126.9 ± 18.8	123.5 ± 17.4	0.171	135.7 ± 15.7	127.3 ± 15.1	0.106
DBP-Base, mmHg	72.7 ± 13.9	69.6 ± 12.0	0.085	78.5 ± 12.4	67.2 ± 13.4	0.009
HR-Base, bpm	75.0 ± 9.7	71.0 ± 10.7	0.008	73.9 ± 8.4	71.1 ± 13.5	0.389
MoCA	21.8 ± 4.4	22.3 ± 4.9	0.513	21.8 ± 3.4	23.38 ± 5.0	0.218
MMSE	26.7 ± 2.7	26.9 ± 3.4	0.640	26.9 ± 3.03	28.0 ± 4.5	0.234
NMSS	58.6 ± 49.2	33.3 ± 28.1	0.001	51.3 ± 29.1	52.7 ± 30.6	0.970
PDQ39	53.2 ± 36.9	34.4 ± 25.4	0.002	52.7 ± 30.6	40.9 ± 33.6	0.283
UPDRS-III	35.9 ± 14.9	32.7 ± 15.9	0.186	33.8 ± 16.5	36.4 ± 18.8	0.678

Values are mean ± standard deviation (SD) or median (interquartile range). H & Y stage, Hoehn and Yahr stage; LEDD, levodopa equivalent daily dose; AHD, antihypertensive drugs; CCB, calcium channel blocker; ACEI, angiotensin-converting enzyme inhibitor; ARB, angiotensin receptor blockers; SBP-base, systolic blood pressure in the supine position; DBP-base, diastolic blood pressure in the supine position; HR, heart rate; MoCA, montreal cognitive assessment; MMSE, mini-mental state examination; PDQ-39, Parkinson's disease questionnaire 39; NMSS, non-motor symptoms scale; UPDRS, unified Parkinson's disease rating scale.

**Figure 1:**
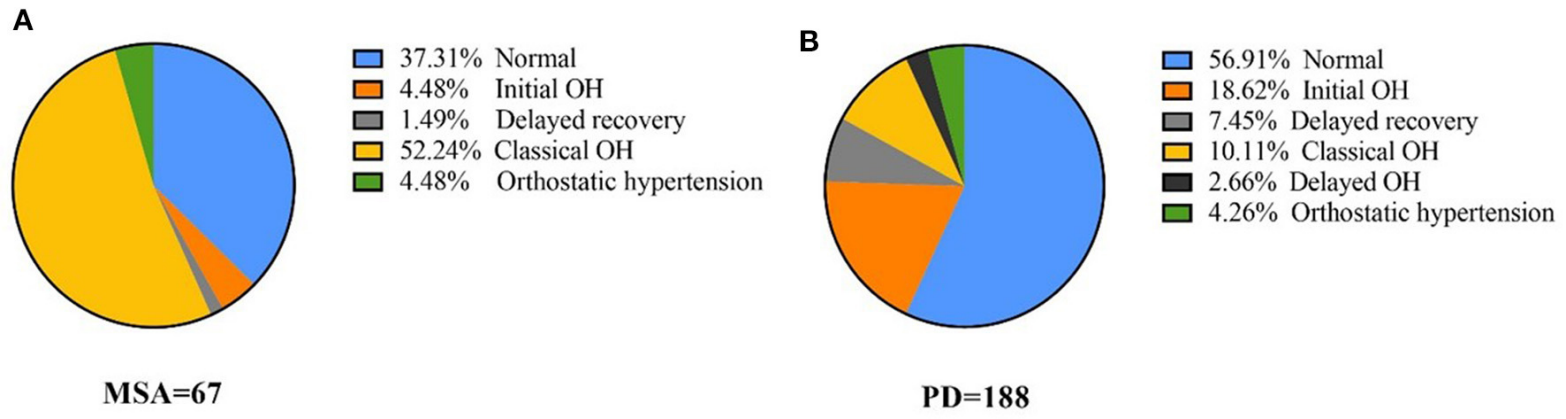
The proportion of orthostatic hypotension and all its variants in MSA and PD **(A)** proportion of blood pressure of different OH types in MSA, **(B)** proportion of blood pressure of different OH types in PD.

All participants were diagnosed according to the results of the active standing test. The component ratio of OH was different between PD and MSA (Pearson χ*2* test: *P* < 0.001), as shown in Figure 1. In total 4.48% of MSA patients had IOH, 1.49% of Delayed recovery and 52.24% of COH. In PD, 18.62% patients had IOH, 7.45% had Delayed recovery, 10.11% had COH and 2.66% had delayed OH. COH (52.24%) was the most common type of OH in MSA, whereas IOH (18.62%) was the most common type of OH in PD. The PD-COH (*n* = 19) was older (67.68 ± 13.09 vs. 58.20 ± 13.62) than PD-IOH (*n* = 35). After controlling age as a covariant, the other demographic and clinical data were still not different between the PD-COH and the PD-IOH groups. There was no difference in BP between MSA-P (*n* = 40) and MSA-C (*n* = 27) except for the cognitive aspect (MoCA, 22.68 ± 3.02 vs. 20.42 ± 5.67, *P* = 0.040; MMSE, 27.30 ± 2.09 vs. 25.73 ±3.29, *P* = 0.021) which was associated with education (F = 3.385, *P* = 0.010; F = 4.861, *P* = 0.001) not the subtype of MSA (F = 0.120, *P* = 0.730; F = 0.339, *P* = 0.563) from Multi-factor analysis of variance.

**Figure 2:**
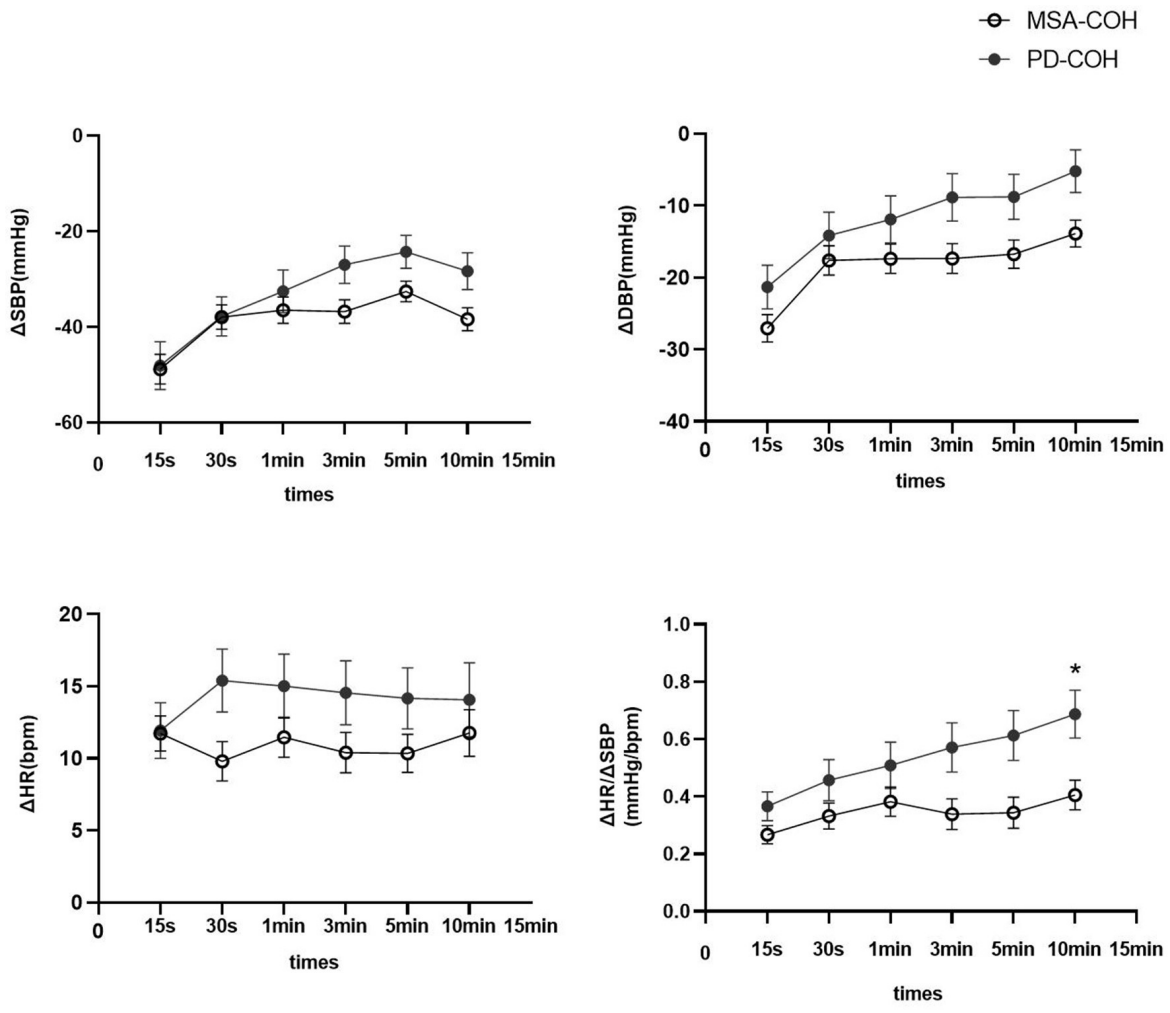
ΔSBP, ΔDBP, ΔHR, and ΔHR/ΔSBP in PD and MSA groups with COH. Vertical lines are standard deviation. MSA-COH had lower ΔSBP, ΔDBP, ΔHR, and ΔHR/ΔSBP than PD-COH. Statistical significance: *P < 0.05.

Moreover, 33 patients with MSA and 13 with PD had COH without severe intracranial or extracranial artery stenosis (Table 1). Patients with MSA-COH had a shorter disease duration than those with PD-COH (2.3 ± 1.7 years vs. 5.8 ± 3.7 years, *P* = 0.006). The SBP (135.70 ± 15.68 mmHg vs. 127.31 ± 15.14 mmHg, *P* = 0.106), DBP (78.45 ± 12.36 mmHg vs. 67.15 ± 13.39 mmHg, *P* = 0.009), and HR (73.94 ± 8.39 bpm vs. 71.08 ± 13.52 bpm, *P* = 0.389) at baseline were higher in patients with MSA-COH than in patients with PD-COH. To determine factors related to higher SBP, DBP, and HR, multiple linear regression analyses were performed. We found SBP was only associated with hypertension and HR was only associated with MSA. DBP was associated with hypertension and MSA. They were not associated with LEED and diabetes. Additionally, patients with MSA-COH had a consistently greater decrease in BP and less increase in HR than patients with PD-COH (Figure 2).

**Figure 3:**
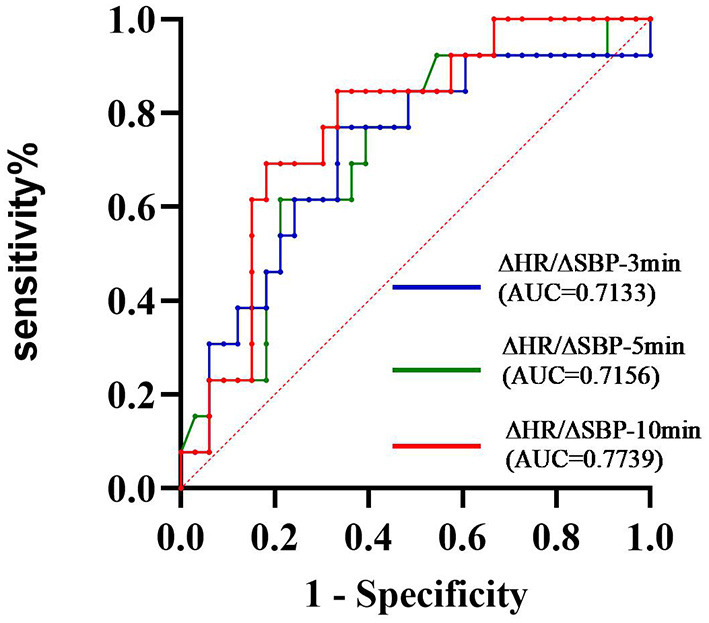
ROC curve showing the sensitivity and specificity of the ΔHR/ΔSBP ratio to distinguish between MSA and PD ΔHR/ΔSBP-10 min was considerably the best index (AUC = 0.7739).

A summary of repeated ANOVA for ΔSBP, ΔDBP, ΔHR, and ΔHR/ΔSBP measurements across diseases and time points is presented in Table 2. The ΔHR/ΔSBP ratio was higher in patients with PD-COH compared to those with MSA-COH (Figure 2). After adjustment for age and disease duration, PD-COH had significantly higher ΔHR/ΔSBP-10 min ratio (0.73 ± 0.19 vs. 0.44 ± 0.29, *P* = 0.031) than MSA-COH. The ΔHR/ΔSBP-10 min ratio had a considerably sensitivity and specificity to discriminate between patients with MSA-COH and PD-COH (AUC = 0.77, 95% CI: 0.63–0.91, *P* = 0.0042, Figure 3). A ΔHR/ΔSBP-10 min value of 0.517 was established as the best cut-off value, with 67% sensitivity and 84% specificity.

**Table 2:** Summary of repeated ANOVA for ΔSBP, ΔDBP, ΔHR, and ΔHR/ΔSBP measurements across MSA and time points.

***N =* 46**	**MSA**		**Time**		**Interaction**	
	***F*** **Value**	**P-value**	***F*** **Value**	**P-value**	***F*** **Value**	**P-value**
ΔSBP, mmHg	2.076	0.157	16.650	<0.001	1.743	0.165
ΔDBP, mmHg	4.260	0.045	22.567	<0.001	0.986	0.407
ΔHR, bpm	2.177	0.147	0.412	0.744	1.430	0.237
ΔHR/ΔSBP, mmHg/bpm	5.571	0.023	10.331	<0.001	2.782	0.032

MSA, multiple system atrophy; Δ, change with respect to basal values; SBP, systolic blood pressure; DBP, diastolic blood pressure; HR, heart rate; n, number of patients; bpm, beats per minute.

## 4. Discussion

In this study, IOH was more prevalent than COH among patients with PD (18.62 vs. 10.11%) and COH (52.24%) was the most common type of OH in MSA. Furthermore, as the standing time increased, the changes in SBP and DBP gradually decreased and were more noticeable in patients with PD-COH than MSA-COH. A ΔHR/ΔSBP ratio at 10-min in the standing position discriminated patients with MSA-COH from those with PD-COH with good sensitivity (67%) and specificity (84%).

The prevalence of OH in PD reportedly ranged from 9.6 to 64.9% (14). Our patients were mainly from wards and the BP was measured by non-invasive continuous blood pressure monitoring which may lead to variation in the prevalence rate of OH. The magnitude of the initial BP reduction is greater for the active standing test than for the passive tilting test (15), and IOH is only associated with active standing. In contrast, passive tilting has no diagnostic value (16). From this, IOH was more to be detected in our study. Van Twist stated that delayed recovery from orthostatic BP changes was associated with an increased risk of falls, cognitive impairment, and frailty, but not the presence of IOH itself (17). However, no significant difference among these various hypotension types was observed in patients with PD. Indeed, OH impairs daily activities and increases the risk of falls and cognitive impairment (18, 19). We also observed that the whole MSA group had high PDQ-39 and NMSS scores in this study, which predicted worse quality of life.

Delayed OH, a mild form of neurogenic OH, with parasympathetic impairment may turn out to be an early biomarker of synucleinopathy (20). A retrospective study of 373 patients with MSA found that 18% of those were diagnosed as having delayed OH which (compared with a delayed onset after 3 min) was more often reported in patients with OH symptoms, more severe disability and in those with “probable” MSA (21). Yoo SW found that 22.5% patients diagnosed with early drug-naïve PD had delayed OH (22). In our study, the percentages of delayed OH in MSA and PD are 0 and 2.66% respectively which are much lower than the above studies. We suspected that it may be due to small sample size, different races, and different duration of disease.

Veera observed cardiac output (CO) and vascular resistance (VR) with (pre) syncope in a real-world clinical setting and discovered that CO seemed to be the primary determinant in the pronounced BP fall upon standing in IOH, impaired increase in VR was the main determinant of delayed BP recovery, and COH could be reflected in the wide range of CO and VR changes (23). Various types of OH have often been neglected in previous PD reports. Haensch suggested that not all components of the autonomic nervous system are uniformly affected simultaneously in the course of disease progression in PD (24), similar to Braak's staging of the brain pathology of sporadic PD. Hence, different degrees of central and peripheral damage were mixed leading to different duration and amplitude of OH.

Patients with MSA showed higher SBP, DBP, and HR than those with PD in the supine position—in the whole group and separate subgroups. Ambulatory BP monitoring recorded a higher nocturnal HR in MSA patients than in PD patients (25). Usually, parasympathetic activity prevails over the sympathetic nervous system in controlling HR at rest. Previous studies have observed that the parasympathetic cholinergic component of the autonomic nervous system in PD-OH patients was preserved (26). We speculated that the higher HR was due to the deficient vagal activity in MSA. It has been postulated that the etiology of supine hypertension in MSA could be due to residual sympathetic tone in peripheral nerve fibers and increased sensitivity to norepinephrine on the postsynaptic membrane (27). In addition, the elevation of renin and angiotensin levels compensates for chronic OH. Higher SBP and DBP is a precursor to supine hypertension.

Patients with MSA had a smaller rise in HR during standing than patients with PD and was also observed in the head-up tilt test (28, 29). However, several studies have reported the opposite conclusion that HR response remains high in patients with MSA (5, 30). These two paradoxical results may be attributed to different illness severity, standing-test methods, and large individual diversity in PD.

The decrease in BP in MSA patients lasted for a long time with a large amplitude, which is the same as in previous studies where OH was more prominent in MSA than in PD (26). This could be because the cardiac response during orthostatic stress is lower in MSA than in PD. The OH of MSA is related to the degeneration of preganglionic autonomic neurons of the brainstem and spinal cord, especially the rostral ventrolateral medullary neurons. In PD, OH is likely related to the loss of peripheral noradrenergic innervation of cardiovascular targets (31). The BP change caused by a central lesion may be more severe and synchronous than that caused by a peripheral nerve injury.

The tilt table test seems more capable than the Valsalva maneuver in identifying cardiovascular abnormalities in Parkinsonian syndromes patients (32). Nocturnal HR and nocturnal decline in HR can discriminate MSA from PD with acceptable accuracy (27). Vallelonga et al. confirmed that the ΔHR/ΔSBP ratio is a simple and accurate test to suggest an autonomic failure in PD patients with OH, compared with reverse dipping and increased diurnal SBP standard deviation (33). The ΔHR/ΔSBP-3 min ratio did not differ between the central and peripheral groups (5). We selected PD-COH and MSA-COH without intracranial or extracranial artery stenosis and observed that ΔHR/ΔSBP at 10 min significantly differed between the two groups. When we compared ΔHR/ΔSBP at different timepoints (3 vs. 10 min) in the whole population, no difference was found. For above reasons, we recommend prolonged active standing for 10 min when a patient has COH.

This study had some limitations. First, the diagnosis of PD and MSA lacked pathological confirmation and the patients who were unable to stand for 10 min were excluded. Second, we did not include a comparison group of normal controls, which would have made the study more comparable. Third, PD patients were at an earlier stage than MSA in this study and the change of BP in two groups may look more similar in a more advanced phase.

In conclusion, IOH is a common phenomenon in PD. More attention should be paid to the outcome and prognosis of various types of IOH, which may aid the understanding of the process and mechanism of autonomic failure. ΔHR/ΔSBP-10 min is readily available in clinical practice, and further studies are needed to clarify whether it is a reliable parameter for discriminating MSA from PD.

## Data availability statement

The original contributions presented in the study are included in the article/supplementary material, further inquiries can be directed to the corresponding author.

## Ethics statement

The studies involving human participants were reviewed and approved by the Ethics Committee of the Capital Medical University Xuanwu Hospital. The patients/participants provided their written informed consent to participate in this study.

## Author contributions

JZ analyzed the data, interpreted the data, and drafted the manuscript for intellectual content. XX and HS analyzed the data and interpreted the data. SM, BX, YX, and EX revised the manuscript. All authors contributed to the study conception and design.
